# Treatment and Toxicity Considerations in Tuberculosis: A Narrative Review

**DOI:** 10.7759/cureus.62698

**Published:** 2024-06-19

**Authors:** Nicholas T Jones, Raegan Abadie, Camryn L Keller, Kamryn Jones, Lloyd F Ledet III, Julia E Fox, Vincent G Klapper, Pooja Potharaju, Harish Siddaiah, Adam M Kaye, Sahar Shekoohi, Alan D Kaye, Giustino Varrassi

**Affiliations:** 1 School of Medicine, Louisiana State University Health Sciences Center, Shreveport, USA; 2 Department of Internal Medicine, Louisiana State University Health Sciences Center, Shreveport, USA; 3 Department of Anesthesiology, Louisiana State University Health Sciences Center, Shreveport, USA; 4 Department of Pharmacy Practice, Thomas J. Long School of Pharmacy and Health Sciences, University of the Pacific, Stockton, USA; 5 Department of Pain Medicine, Paolo Procacci Foundation, Rome, ITA

**Keywords:** ethambutol, pyrazinamide, rifampin, isoniazid, side effects, mycobacterium tuberculosis

## Abstract

Tuberculosis remains one of the most significant bacterial infections plaguing the medical community worldwide. The bacteria *Mycobacterium tuberculosis* retains the ability to manifest as an active infection, latent infection, miliary infection, or reactivation of latent infections in times of immunosuppression. Therefore, the medication regimen to treat the condition revolves around four medications, each with a mechanism that targets a different part of the bacteria. Isoniazid weakens the cell wall but produces neuropathy and hepatotoxicity as side effects. Rifampin interrupts protein synthesis but creates the opportunity for many drug-to-drug interactions and red-orange discolorations as side effects. Pyrazinamide is poorly understood, but it is believed to acidify the internal environment of the bacteria, with gout exacerbations and arthralgias as major side effects. Ethambutol also works as a bacteriostatic medication to interrupt the cell membrane; however, its mechanism is poorly understood. The most concerning side effect is optic neuropathy. The unfavorable side effect profile for tuberculosis treatment may contribute to the higher rates of medication noncompliance with therapy and needs to be addressed in the future.

## Introduction and background

Tuberculosis (TB) remains a constant threat to public health as it is highly infectious and the leading cause of death contributed to a single infectious agent worldwide [1]. In South and East Asia and the Pacific regions, they suffer the highest economic losses, while sub-Saharan Africa experiences the highest loss of life expectancy with respect to TB [2]. Infection with TB typically results in a latent and chronic carrier state in an estimated one-third of the population, or approximately two billion people [3]. The disease TB is mostly caused by the bacteria *Mycobacterium tuberculosis*, with other bacteria in the genus causing a minority of TB infections. TB is a highly infectious disease that is primarily spread through airborne transmission of infectious droplets that remain in the air for up to hours at a time when a patient with active TB coughs and a susceptible patient inhales the particles, with other routes of transmission as a minor means of transmissibility [4]. Once infected with the bacteria, the microbe replicates intracellularly within the immune system's macrophages, crosses the alveolar basement membranes within the lungs, and could disseminate into the rest of the body. After exposure, there are multiple outcomes of infection. Those with a weak immune system are at risk of getting a miliary infection, where the infection spreads to multiple organ systems. Immunocompetent patients can clear the infection completely or arrest bacterial growth and maintain a latent infection. Latent infections are relatively common, with the World Health Organization (WHO) estimating that approximately one-third of the world’s population has a latent TB infection, with 5%-10% likely to have an episode of reactivation TB. Reactivations typically occur when the immune of the host becomes weaker, typically when patients age or have an HIV coinfection [5,6]. Diagnosis of latent TB in patients can be achieved via a tuberculin skin test, also known as a purified protein derivative (PPD). This test requires a registered administrator, typically a nurse, to inject an antigen to the *Mycobacterium* under the skin, with a positive test being a >5 mm induration within 48 to 72 hours of injection. Since a PPD lacks sensitivity and specificity, it is useful to screen for exposure before moving on to more expensive and specific tests [7]. The PPD test works through a type-IV hypersensitivity that allows the adaptive immune system of a person, specifically T-cells, to react [8]. A positive result simply means that this patient has been exposed to the antigen at some point in their lives and does not confirm a diagnosis of TB until further blood tests, such as the QuantiFERON-TB Gold Plus test, can be performed. The QuantiFERON test is commonly performed in developed countries, such as the United States, and with sensitivity and specificity of 83.3% and 90.1%, respectively, it makes an excellent confirmatory test [9,10]. Interferon-gamma release assay (IGRA) is another TB blood test that assists with diagnosis when a person has signs of TB.

Once a diagnosis is confirmed, resistance testing needs to rule out any drug-resistant microbes before treatment to ensure that the medical healthcare system prevents the creation of more pan-resistant strands of *M. tuberculosis*, such as those already resistant to isoniazid or rifampin [11]. Currently, the treatment after the diagnosis of TB with no resistance is an intensive four-medication phase for two months consisting of isoniazid, ethambutol, rifampin or rifapentine, and pyrazinamide followed by a longer continuation phase with only two medications [12]. Since the creation of this regimen of medication, the lives of many patients have been saved. However, there are no medications without risk, and each of the four TB medications comes with its array of unique side effects. Due to the high rate of side effects, it is one of the leading causes of medication poor adherence or discontinuation in patients who are being treated for TB infection. With high rates of discontinuation, there is a higher chance for drug resistance to develop, contagion, and reinfection [13]. This review serves as a survey of the dosage, contraindications, and side effects of each medication used in the treatment of drug-sensitive TB.

## Review

Methods

A comprehensive systematic search on PubMed was conducted utilizing broad terms to encompass all relevant topics without premature exclusions. All publications were searched for in English and filtered to include clinical controlled trials since 2022 for recent research. The search strings included the name of each medication, “isoniazid,” “rifampin,” “pyrazinamide,” OR “ethambutol,” AND "side effects." After the initial search, the articles that quantified the side effect rates of each drug were selected. These new studies have been incorporated into this review.

Isoniazid

Dosage and Contraindications

Isoniazid was first used to treat TB patients in 1952 and quickly decreased the number of TB patients within two years. Isoniazid’s effectiveness at treating patients with TB is by passively diffusing into *M. tuberculosis*, which then the prodrug of isoniazid is activated by KatG catalase-peroxidase to form an isoniazid-NAD+ adduct. This new form of isoniazid provides its lethality to *M. tuberculosis* because of its inhibitory effects on mycolic acid and cell wall production. Isoniazid is still the first-line antibiotic for TB patients taking rifampin, ethambutol, and pyrazinamide [14].

Isoniazid can be administered orally, intramuscularly, or intravenously as a slow five-minute bolus in 25 mL of normal saline. The oral tablet doses for adults are 50, 100, or 300 mg, or solution (50 mg/5 mL) with a dosage of 4-6 mg/kg with a typical daily dose of 300 mg. However, administering intravenously or intramuscularly for critically ill patients is either 5 mg/kg (300 mg typically) daily, five days per week, or 15 mg/kg (900 mg normally) once, twice, or thrice per week. Patients are recommended not to take isoniazid with food because of the risk of decreasing isoniazid’s bioavailability and absorption. If taken with food, isoniazid can be reduced to hydrazine [14].

Side Effects

Isoniazid’s mechanism of action first involves inactivation via acetylation of its hydrazine group by phase II enzyme N-acetyltransferase 2 in the liver and intestines. With 32 known genetic variability in N-acetyltransferase 2, different metabolism rates of isoniazid among individuals and different isoniazid-induced toxicity in TB patients, specifically hepatotoxicity, can be observed. Previous literature shows that TB patients with a slow acetylator phenotype of N-acetyltransferase 2, compared to the rapid acetylator phenotype of N-acetyltransferase 2, have a higher risk of anti-TB drug-induced hepatotoxicity. Slow acetylators can accumulate isoniazid toxic metabolites by not rapidly acetylating them. Hepatic injury can also occur with these slow acetylators because unacetylated isoniazid can be hydrolyzed by amidase into hydrazine, leading to hepatic injury. Therefore, the slow N-acetyltransferase 2 acetylators do not acetylate isoniazid fast enough, which can lead to the accumulation of its intermediates that can be toxic.

With isoniazid being so widely used for the treatment of TB patients, the toxic side effects should be minimized. Personalized medication may be the solution to avoid isoniazid-induced drug injury, specifically hepatotoxicity. This is because of all the variability of polymorphisms affecting the metabolism of isoniazid. When comparing the two different polymorphisms of N-acetyltransferase 2, rapid and slow, it was found that altering the dosages can minimize side effects. For example, individuals with rapid acetylation polymorphism of N-acetyltransferase 2 can be given a higher dose or tablet of isoniazid without causing side effects. However, slow acetylation polymorphisms of N-acetyltransferase 2, a lower dosage of one-half the standard isoniazid dosage, were recommended to reduce the isoniazid side effects on the liver. The two different polymorphisms of N-acetyltransferase 2 vary among other ethnicities, so with personalized medication, genotyping can be done to determine isoniazid's effective and sustainable dosage while minimizing hepatic toxicity [14].

Isoniazid has monoamine oxidase (MAO) inhibition activity, so dietary and drug restrictions are essential. Antidepressants and other medications with serotonergic mediated or modulated pharmacology and MAOI activity, such as the oxazolidinone class of antibiotics (e.g., linezolid, tedizolid), need to be considered related to potential drug-drug interactions. In this regard, avoidance of foods that can provoke a tyramine reaction is necessary [12]. Tyramine consumption can lead to the release of norepinephrine (NE), and it is found in high concentrations in foods, such as aged cheese and red wine, including chianti, skipjack, tuna, fermented meats, sausages, salami, cacciatore, and mortadella. Under normal conditions, NE cannot accumulate to toxic levels related to the presence of MAO-A, an enzyme that degrades neurotransmitters, including NE.

Isoniazid dosage administration of 20 mg/kg will compete with pyridoxine (vitamin B6), which can cause a bilateral reduction in visual acuity and optic disc swelling bilaterally, appearing as bitemporal hemianopia scotomas [15]. Since isoniazid reduces pyridoxine activity, it is essential to maintain this vitamin consumption as both prevention and acutely, if needed, as an antidote in overdose. Clinical pyridoxine depletion can contribute to neuropathic symptoms as well.

Isoniazid’s ability to reduce gamma-aminobutyric acid (GABA) synthesis can result in neurotoxic effects, including convulsions/seizures, neuropathy, encephalopathy, optic neuritis and atrophy, memory impairment, and toxic psychosis. Other potential adverse reactions are agranulocytosis, rhabdomyolysis, pellagra, hyperglycemia, metabolic acidosis, and gynecomastia [14]. Isoniazid can cause psychosis in children, including increased aggression, the tendency to bite others, and eating mud, wood, clothes, etc. Isoniazid-induced psychosis is a rare adverse effect with an incidence of 1%-2% [16].

In a study by Lv and Tang (2024), two participants in the control group had liver function abnormalities (4.55%), two had gastrointestinal reactions (4.55%), and three had rashes (6.82%). In the intervention group, two participants had liver function abnormalities (3.57%), three had gastrointestinal reactions (5.36%), and three had rashes (5.36%). The intervention group’s total incidence rate of adverse reactions was 14.29% for eight participants compared to the control group’s rate of 15.91% for seven participants. No significant difference existed between the groups in the total incidence rate (p > 0.05) [17].

Rifampin, rifabutin, and rifapentine

Rifampin, rifabutin, and rifapentine are all derivatives of rifamycin, an antibiotic obtained from the fermentation of *Streptomyces mediterranei*. It works by disturbing the interaction between ribosome and RNA, and this interaction is essential for the translation of RNA into protein. This prevents the growth of dividing cells, halting *M. tuberculosis* replication within host cells. Rifampin is highly effective in treating TB if given for a sufficient length of time at an adequate dose, both of which have been shown to have a higher compliance rate [18]. Rifampin, rifabutin, and rifapentine have similar mechanisms of action; however, rifampin is the most potent inducer of the cytochrome P-450 (CYP-450) metabolic enzymes. This causes a variety of drug-drug interactions and increased drug concentrations in patients taking other medications metabolized by these enzymes [19].

Dosage and Contraindications

Rifampin and rifabutin have very similar structures and have both been effective with daily dosing. However, rifabutin has a longer half-life of two to three hours, which means it can be given at a lower dose [20]. Rifapentine has a significantly longer half-life of 14-15 hours, so it can be administered once weekly as opposed to daily. This has been shown to increase patient compliance [21]. Because of the induction of the CYP-450 system, the largest contraindication for taking any of the rifamycin derivatives is if they are taking any of these drugs metabolized by CYP-450 [22]. These medications include HIV-related protease inhibitors, antifungal drugs, and immunosuppressants such as tacrolimus and cyclosporine [23].

Side Effects

By increasing the activity of the CYP-450 enzymes, the medications metabolized by the CYP-450 system will have decreased activity. This occurs because the drugs are being processed by the CYP-450 enzymes into excretable compounds to rapidly remove them from the body, weakening their activity in tissue. Rifampin has been shown to reduce the activity of HIV protease inhibitors by up to 44%, which can lead to treatment failure in these patients unless changes to medication doses are made [23]. However, this is not seen in patients taking rifabutin due to it having less of an effect on CYP-450 enzymes [24]. Additionally, rifapentine is not recommended in the treatment of TB in HIV patients because a study shows that doing so increases patient resistance [23]. Antifungals, specifically azole antifungals that work by inhibiting the CYP-450 enzyme necessary for ergosterol synthesis, are also rapidly metabolized when combined with rifampin and rifabutin [19]. One study showed that after receiving rifampin for multiple weeks, the elimination rate of fluconazole increased by 39%. The dose of fluconazole is advised to be increased by 30% to achieve adequate treatment of infections with fungi such as *Cryptococcus neoformans* [23]. Immunosuppressants, such as tacrolimus, have also been shown to interact with both rifampin and rifabutin, and the rifamycin drugs can cause an increase in clearance of over 50% along with a significantly decreased bioavailability. It has been determined that the tacrolimus dose must be increased to 10-fold to maintain concentrations within the serum [19]. This reduction in tacrolimus activity even occurred when given concomitantly with CYP-450 inhibitors such as fluconazole and clarithromycin [23]. Cyclosporine is another immunosuppressant that interacts with these drugs; however, doses must only be increased up to three-fold to maintain pre-rifampin drug concentrations. The increased metabolic functioning of the liver can reduce the reliability of oral or other systemic hormonal contraceptives necessitating the need for alternative contraceptive measures [23].

One study conducted in 2022 compared the effects of high-dose versus low-dose rifampin in the context of HIV-associated tuberculous meningitis. Transaminitis, defined by Division of AIDS (DAIDS) criteria for grades 3 and 4, was of particular interest. Utilizing a dosage regimen of 35 mg/kg daily, the study found that out of the 16 individuals in the treatment group, two cases, equating to 13%, exhibited transaminitis. Notably, one patient displayed improvement to grade 2 but could not resume rifampicin due to medical constraints, while another patient experienced complete resolution and was transitioned to Rifafour from rifampicin. These occurrences were observed on days 16 and 6, respectively. The statistical analysis yielded a p-value of 0.109, underscoring the need for further research to ascertain the significance of these results in informing clinical practice and treatment protocols [25]. In a study conducted in 2023 comparing normal dosing and triple dosing of rifampin for TB, distinct patterns of adverse effects emerged. Among patients receiving the normal dose of rifampin, 16.9% (11 out of 65) reported experiencing any adverse effect, with only 3% (2 out of 65) encountering higher-grade adverse effects, primarily based on the level of elevation of alanine aminotransferase (ALT). Contrastingly, among those administered the triple dose, a higher incidence of adverse effects was noted, with 29% (18 out of 62) reporting any adverse effect. Moreover, 17.7% (11 out of 62) experienced higher-grade adverse effects, and notably, 6.5% (4 out of 62) suffered from hepatotoxicity. These findings underscore the heightened risk of adverse effects associated with triple dosing compared to normal dosing of rifampin in TB treatment, warranting careful consideration in clinical decision-making [26]. A study conducted in 2023 investigated the comparative efficacy and adverse effects of high-dose versus normal-dose rifampin for brucellosis treatment. The study found that while there were significant rates of adverse effects notable in both groups. High-dose rifampin, administered at 900-1,200 mg/day, and normal-dose rifampin at 600mg/day were associated with adverse effects, including nausea, rash, vomiting, and transaminitis. Interestingly, a lower proportion of patients in the high-dose group (5%) experienced no clinical response or improvement compared to the normal-dose group (18.33%). The difference in clinical response between the two groups was statistically significant, with a p-value of 0.02. Despite an overall higher occurrence of adverse effects in the high-dose group, the difference between the groups was not statistically significant. These findings suggest that while high-dose rifampin may offer some advantages in terms of clinical response, careful consideration of adverse effects is warranted when determining the appropriate dosage regimen for brucellosis treatment [27].

Pyrazinamide

Pyrazinamide is an anti-TB medication that acts as a nicotinamide analog [28]. As it enters *M. tuberculosis*, through passive diffusion, it is converted from the prodrug to its active form, pyrazinoic acid, by the pncA gene encoding pyrazinamidase [29]. Pyrazinamide is utilized as a secondary treatment for multidrug-resistant TB but can be incorporated following proper drug susceptibility testing. Typically, it is administered during the initial two months of therapy for standard drug-sensitive TB [30]. Incorporating pyrazinamide into rifampin-based therapy appeared to shorten the treatment duration by three months, from six to nine months. Its inclusion in the regimen also decreased the TB relapse rate in organisms with low metabolic and replication rates [31].

Although its mechanism of action is still being studied, it is thought that pyrazinoic acid accumulates in bacterial cells, creating an acidic pH [29]. This could lead to an inhibition of the cells’ enzymes and lead to the depletion of the proton motive force across the membrane, thus inhibiting translation [29]. It is particularly efficient in killing non-replicating bacteria, yet persistent and resistant to other TB drugs. Rendering it bactericidal and bacteriostatic. Pyrazinamide demonstrates heightened activity in acidic environments, particularly in cases with cavitary lesions in active TB due to its ability to penetrate lung lesions and exert an intracellular sterilizing effect within granulomas is noteworthy [30]. Generally, mutations in the pncA gene are the primary mechanism of resistance, although other genes, such as the RpsA or PanD genes, have also been implicated in some resistant strains [29].

Standard dosing for pyrazinamide in adults is a daily therapy of 15-20 mg/kg PO quay or a twice-weekly therapy of 50 mg/kg PO twice weekly (not to exceed 2 g/dose for adults) [32]. It is only given in addition to rifampin or isoniazid, not as a sole medication. A study analyzing laboratory, animal, and clinical trials regarding pyrazinamide dosage and its effect on microbial response found a clear link between a dose range of 15-50 mg/kg/day and a decrease in bacterial count ranging from 0.50 to 27.7 log_10_ CFU/mL [30]. In mouse models, higher pyrazinamide doses led to greater reductions in bacterial load, and human trials also showed that increased drug exposure correlated with improved efficacy in reducing bacterial burden [30]. Contraindications include those with preexisting liver or kidney injury due to the increased retention of toxic pyrazinamide metabolites [32,33].

Side Effects

Other side effects include gout attacks, rash, photosensitivity (after sun exposure), arthralgias, and gastrointestinal upset [32]. The major side effect of pyrazinamide is hepatoxicity, displayed by increasing elevations of serum aminotransferase, with the onset of liver injury occurring four to eight weeks after beginning medication [28]. This drug-induced liver injury occurs in less than 1% of patients and is correlated with longer uses and higher dosages of the drug [34]. Additionally, differences in sex, HIV co-infection, and hepatic and renal function can influence the pharmacokinetics of pyrazinamide among patients [30]. Given that pyrazinamide undergoes hepatic metabolism via microsomal deamidase to become active, variations in enzyme function levels may result in diverse treatment responses across patients [30]. Pyrazinamide is considered more hepatotoxic than isoniazid or rifampin, but the mechanism is poorly understood because it is usually used with other anti-TB drugs, which are also hepatotoxic [34]. Yet combination therapy with rifampin and pyrazinamide is no longer used due to its tendency to cause severe liver injury. There is some evidence to suggest that metabolites from the activated form of pyrazinamide contribute to hepatotoxicity, causing acute hepatitis and leading to portal and lobular hepatocellular necrosis [28]. In patients with prior liver or kidney disease, there is a reduction in the kidney clearance of the drug and a prolonged half-life, further contributing to liver injury and requiring an adjusted dose [34]. Liver toxicity is also dose-dependent on doses above 40 mg/kg, requiring close monitoring of liver enzymes and kidney function [34].

Pyrazinamide can also cause an increase in urate retention, leading to decreased renal clearance of uric acid due to pyrazinoic acid being oxidized by xanthine oxidase into 5-hydroxy-pyrazinoic acid [35]. This can precipitate acute gout attacks that can be treated with hydration in patients who normally do not have uric acid imbalances [35]. Those who do suffer from recurrent gout attacks can be treated with nonsteroidal inflammatory drugs, colchicine, corticosteroids, or xanthine oxidase inhibitors such as allopurinol or febuxostat [35].

Ethambutol

Ethambutol began being used for treating and managing TB in patients in the 1960s. Ethambutol is a bacteriostatic drug that inhibits arabinogalactan production in the cell wall. With ethambutol specifically, only its D isotype form is used since the L form proved toxic. Ethambutol is best when used in a combination drug with other anti-TB drugs, like isoniazid, and avoided in solo treatment. Ethambutol can have synergistic effects for *M. tuberculosis* when used in conjunction with isoniazid, as both medications harm the bacterial cell wall independently.

Oral dosing of ethambutol for adults is 100 mg or 400 mg, but the oral dosage is recommended for children as 15-20 mg/kg of their body weight. Ethambutol is one of the first lines of treatments for TB when used with isoniazid, rifampicin, and pyrazinamide for two months, and then isoniazid and rifampicin and/or ethambutol for the following four months [14].

Side Effects

One of the main side effects of treatment with ethambutol is the loss of visual acuity. Examples of optic side effects due to ethambutol are optic neuropathy, optic neuritis, retrobulbar neuritis, and peripheral neuropathy. Ethambutol also has non-optic side effects, including hepatotoxicity, numbness and tingling of extremities, mental confusion, disorientation, hallucinations, and psychosis. Ethambutol-induced optic side effects are seen more often in patients with pre-existing lower renal function due to the liver excretion method used for ethambutol. Therefore, patients should be screened before therapy is started. Patients also need to be screened before starting therapy with ethambutol for possible contraindications, like those patients who are incapable of noting visual symptoms such as those with dementia, mental retardation, and children. Ethambutol should not be administered to those with pre-existing ophthalmological diseases.

Ethambutol-induced optic neuropathy is a common side effect of TB treatment but can be irreversible and preventable with regularly scheduled screenings by ophthalmologists. Therefore, before starting a patient on ethambutol for the treatment of TB, the entire interprofessional team should be consulted [36].

Multiple studies have studied the effects of ethambutol on optic toxicity. Based on one study, elderly males were known to be most affected by ethambutol-induced toxicity by having the highest decrease in retinal nerve fiber layer thickness. One significant factor that plays into the effect of ethambutol-induced visual disturbance is age. As one age, the risk of visual disturbances increases due to aging compromising renal function. Visual field defects appeared in those patients with increased dosages and timeframes of treatment of ethambutol.

Patients commonly suffer from thinner peripapillary retinal nerve fiber layer thickness. Optic toxicity due to ethambutol is due to its zinc-chelating properties and metabolite on mitochondrial enzymes. Ethambutol at a dose over 20 mg/kg/day can injure fibers in the papillomacular bundle, which is important with central vision. Spectral-domain (SD) optical coherence tomography can be used to rapidly discover visual defects in patients on ethambutol. Ophthalmologists and the entire professional team should be monitoring patients’ visual acuity, color vision, visual fields, retinal nerve fiber layer thickness, and macular thickness all of which can occur due to ethambutol-induced toxicity [15].

In one study, a total of 69 from the initial 80 patients with visual complaints were categorized into two groups, one with ongoing anti-tubercular therapy with ethambutol and the other group with having stopped ethambutol for at least six months. All patients then underwent vision testing. Of the initial 69 patients, 58 patients reported reduced visual acuity. Thirty-three out of 58 patients with reduced visual acuity showed normal optic discs while 25 out of 58 had altered optic discs. In the one group of those who have stopped ethambutol for at least six months, 14 out of the 32 patients had a vision of less than 20/20 and optic disc pallor. In the group that continued to receive ethambutol, 12 of the 15 patients had altered color vision and had vision less than 20/20. Ethambutol toxicity has significant visual side effects, and it is important to establish screening protocols to prevent toxic optic neuropathy (Table 1) [37].

**Table 1:** Recent studies on the side effects of different TB medications

Author (Year)	Groups studied and intervention	Results and findings	Conclusions
Ekqvist et al. (2022) [38]	Hospitalized adults with pulmonary TB were treated with higher doses of rifampin and pyrazinamide and normal doses of isoniazid and ethambutol.	Results are pending.	Higher doses of these two medications shorten the treatment time with a tolerable side effect profile.
Campbell et al. (2020) [39]	Adults with latent positive tuberculosis and indication for treatment, with 3,205 receiving isoniazid and 3,280 participants receiving rifampin therapy.	The adjusted odds ratio for an adverse event for isoniazid and rifampin were 1.8 (95% CI 1.1-3.0) and 1.1 (95% CI 0.6-2.1), respectively.	Rifampin is the safer medication with fewer adverse events for latent TB infection treatment.
Thamineni et al. (2022) [40]	A total of 488 patients received anti-tuberculosis treatment with these four medications.	Subjects reported a 64.8% adherence, and urine studies showed a 53.4% adherence. Side effects comprise 18.6% of the reasons for non-adherence.	Nearly half of patients were nonadherent to their medication regimen, and side effects play a major role in patient adherence as well.

Newer treatment regimens for drug-resistant TB

In 2021, approximately 10.6 million individuals developed TB, with 450,000 new cases of rifampin-resistant (RR) or multi-drug-resistant TB (MDR-TB). The TB cases represented a 3.6% increase in incidence when comparing 2021 to 2020, thus emphasizing the continued presence of TB as a significant danger to the world population [41-43]. Several definitions describe the resistance to TB medications: RR, MDR-TB, pre-extensively drug-resistant TB (pre-XDR-TB), or extensively drug-resistant TB (XDR-TB). MDR-TB has resistance to rifampin and isoniazid, and pre-XDR-TB is an MDR/RR TB-resistant strain, plus resistance to later generations of fluoroquinolones. XDR-TB satisfies the definition of pre-XDR-TB plus resistance to an additional group A medication, which includes bedaquiline or linezolid [41,44]. The 2022 WHO guidelines present two important changes to the treatment of MDR/RR-TB and pre-XDR-TB [45,46]. Based on a multisite, randomized, controlled trial called the Pragmatic Clinical Trial for a More Effective, Concise, and Less Toxic Regimen or TB-PRACTECAL, the effect of a 24-week regimen of only oral medications for pulmonary RR-TB was investigated [47]. The bedaquiline, pretomanid, linezolid, and moxifloxacin (BPaLM) regimen included bedaquiline (a daily dose of 400 mg for two weeks, then 200 mg three times per week for 22 weeks); pretomanoid (a daily dose of 200 mg for 24 weeks), linezolid (a daily dose of 600 mg for 16 weeks, then 300 mg for eight weeks) and moxifloxacin (a daily dose of 400 mg for 24 weeks). It was determined that the BPaLM regimen was more effective than the standard treatment of care (89% compared to 52%) [37], which was typically characterized by a costly nine to 20 months treatment period, up to 20 pills daily, and adverse reactions [47]. In addition, the new six-month regimen was also safer, with less evidence of treatment failure and mortality [47]. For individuals with pre-XDR-TB, the BPaL can be used, excluding monofloxacin [46]. It is also important to note that another trial study (ZeNIX-TB) also supported the 600 mg dose of linezolid instead of a 1,200 mg dose [46,48]. There are restrictions for the administration of bedaquiline, pretomanid, and linezolid (BPaL) or BPaLM. Only adults and children older than 14 years of age can be given the regimens because of limited safety data about the use of pretomanoid in children of a lesser age. Though an individual’s HIV status does not in itself preclude the use of these regimens, caution should be exercised if the CD4 cell count is less than 100 cells/mm^3^. These regimens are also not recommended for miliary, osteoarticular, or central nervous system TB [46].

## Conclusions

TB treatment remains the cornerstone treatment in medicine, as it reduces hospitalizations and is essential in reducing the spread of TB. The reward for using such medical interventions heavily outweighs the risks. Isonizid’s notable side effects include hepatotoxicity and periphery neuropathy, the latter producing a burning sensation in the feet but preventable with pyroxidine supplementation. Despite these, isoniazid remains first-line for *M. tuberculosis*. Rifampin, often admitted after isoniazid, disrupts protein production and significantly induces CYP-450, causing numerous drug-drug interactions. Their interactions include antivirals for HIV, various antifungals, and immunosuppressants, reducing their effectiveness. Additionally, the metabolites created by rifampin metabolism produce a red-orange coloring of urine, which can be concerning for patients. By changing the frequency of medication administration, patient compliance with rifampin has increased. Pyrazinamide works best for persistent infections caused by TB infection. The final medication of TB quad-therapy is ethambutol, which is bacteriostatic; therefore, solo treatment is avoided. The most concerning side effect of ethambutol is optic neuropathy, particularly in patients with decreased renal function. Most of the significant side effects can be prevented by close following, in the case of optic neuropathy, or medical intervention, B6 for isoniazid-induced neuropathy. This side effect reduction is a significant part of patient treatment for TB because patients will opt to cease medications if their side effect profile is too unfavorable. Future research should focus on reducing the side effects of these medications because of the high rate of patients discontinuing the drugs related to side effects, which results in the recurrence of disease.
